# Sparstolonin B Inhibits Pro-Angiogenic Functions and Blocks Cell Cycle Progression in Endothelial Cells

**DOI:** 10.1371/journal.pone.0070500

**Published:** 2013-08-05

**Authors:** Henry R. Bateman, Qiaoli Liang, Daping Fan, Vanessa Rodriguez, Susan M. Lessner

**Affiliations:** 1 Department of Cell Biology and Anatomy, University of South Carolina, School of Medicine, Columbia, South Carolina, United States of America; 2 School of Pharmacy, Nanjing University of Chinese Medicine, Nanjing, China; 3 South Carolina Governor's School for Science and Mathematics, Hartsville, South Carolina, United States of America; Southern Illinois University School of Medicine, United States of America

## Abstract

Sparstolonin B (SsnB) is a novel bioactive compound isolated from *Sparganium stoloniferum*, an herb historically used in Traditional Chinese Medicine as an anti-tumor agent. Angiogenesis, the process of new capillary formation from existing blood vessels, is dysregulated in many pathological disorders, including diabetic retinopathy, tumor growth, and atherosclerosis. In functional assays, SsnB inhibited endothelial cell tube formation (Matrigel method) and cell migration (Transwell method) in a dose-dependent manner. Microarray experiments with human umbilical vein endothelial cells (HUVECs) and human coronary artery endothelial cells (HCAECs) demonstrated differential expression of several hundred genes in response to SsnB exposure (916 and 356 genes, respectively, with fold change ≥2, p<0.05, unpaired t-test). Microarray data from both cell types showed significant overlap, including genes associated with cell proliferation and cell cycle. Flow cytometric cell cycle analysis of HUVECs treated with SsnB showed an increase of cells in the G1 phase and a decrease of cells in the S phase. Cyclin E2 (CCNE2) and Cell division cycle 6 (CDC6) are regulatory proteins that control cell cycle progression through the G1/S checkpoint. Both CCNE2 and CDC6 were downregulated in the microarray data. Real Time quantitative PCR confirmed that gene expression of CCNE2 and CDC6 in HUVECs was downregulated after SsnB exposure, to 64% and 35% of controls, respectively. The data suggest that SsnB may exert its anti-angiogenic properties in part by downregulating CCNE2 and CDC6, halting progression through the G1/S checkpoint. In the chick chorioallantoic membrane (CAM) assay, SsnB caused significant reduction in capillary length and branching number relative to the vehicle control group. Overall, SsnB caused a significant reduction in angiogenesis (ANOVA, p<0.05), demonstrating its *ex vivo* efficacy.

## Introduction

Sparstolonin B (SsnB) is a novel bioactive compound isolated from the plant *Sparganium stoloniferum*, a perennial, aquatic plant grown in North and East China, whose tubers have long been used in Traditional Chinese Medicine (TCM) for the treatment of several inflammatory diseases and as an anti-spasmodic and anti-tumor agent. NMR and X-ray crystallography have identified SsnB as a polyphenol with structural similarities to isocoumarins, a class of compounds that often exhibit anti-coagulant, anti-inflammatory, and anti-tumor properties [1]–[3]. SsnB may hold potential as a safe, non-toxic pharmaceutical agent for the treatment of several pathological conditions. At concentrations up to 100 μM, SsnB does not exhibit cytotoxic effects on various cell types, including mouse peritoneal macrophages, HUVECs, human aortic smooth muscle cells, and monocytic THP-1 cells [3]. Prior research has shown that SsnB exhibits strong anti-inflammatory effects on mouse and human macrophages by selectively inhibiting the inflammatory responses of macrophages to ligands for Toll-like Receptor (TLR) 2 and TLR4. SsnB has also been shown to suppress downstream signaling pathways after TLR2 and TLR4 activation, including MAPK and NF-κB [3]. These findings suggest that SsnB may be an antagonist to TLR2 and TLR4.

Angiogenesis refers to capillary formation from existing blood vessels. Angiogenesis plays an important role in many physiological events in the body, including wound healing, embryogenesis, and female reproductive processes [4], [5]. During these normal processes, angiogenesis is highly regulated. Unregulated, excessive angiogenesis contributes to many pathologies, such as rheumatoid arthritis, psoriasis, retinopathy, and tumor growth and metastasis [6]. Inhibiting pathological angiogenesis may prove to be an effective therapy for these angiogenesis-related diseases.

Angiogenesis is a complex process that occurs in several stages and involves interactions between cells, soluble factors, and extracellular matrix molecules. Endothelial cells play a key role in angiogenesis. Endothelial cells can secrete proteolytic enzymes which break down the basement membrane of an existing blood vessel, allowing the cells to invade the surrounding tissues, migrate in response to an angiogenic stimulus, and proliferate. Growth factors and other soluble proteins in the ECM often facilitate and regulate this process, including vascular endothelial growth factor (VEGF). The endothelial cells form a new lumen, start to secrete extracellular matrix molecules, and ultimately form a new capillary [7]–[9]. Mural cells are also recruited to the site and play an important role in angiogenesis. Angiogenesis may be inhibited at any of these key steps [10]. Targeting endothelial cell proliferation, cell migration, and chemotaxis have shown potential in angiogenesis inhibition.

In the present study, we demonstrate that SsnB inhibits endothelial cell functions related to angiogenesis in several *in vitro* functional assays, and we also show that SsnB inhibits angiogenesis in an *ex vivo* assay. The data suggests the potential of SsnB as an anti-angiogenic agent by showing inhibition of human endothelial cell tube formation and migration. We have also examined the effects of SsnB on endothelial cell gene expression, focusing in particular on pathways related to angiogenesis. In addition, our data show that SsnB arrests endothelial cell division in the G1 phase and downregulates the cell cycle proteins cdc6 and cyclin E2. Furthermore, SsnB caused a significant reduction in blood vessel length and branching number in the chick chorioallantoic membrane assay. Overall, these findings add anti-angiogenic and cytostatic properties to the list of diverse effects exhibited by SsnB.

## Experimental Procedures

### Materials

Sparstolonin B was purified from the plant *Sparganium stoloniferum* according to previously published methods [3]. The purity of SsnB was determined to be greater than 99% by HPLC, and a stability test was utilized to ensure that samples were consistently >99% pure.

*Cell Culture* – Human coronary artery endothelial cells (HCAECs), human umbilical vein endothelial cells (HUVECs), and human cardiac microvascular endothelial cells (HMVECs) were obtained from Lonza (Hopkinton, MA) and cultured on polystyrene, tissue culture-treated petri plates (100×20 mm) coated with 0.1% gelatin. HUVECs, HCAECs, and HMVECs were cultured in endothelial cell medium supplemented with 10% fetal bovine serum (FBS) and endothelial cell mitogen/growth supplement (Biomedical Technologies, Stoughton, MA). The endothelial cell medium was replaced every 2–3 days, and the cells were passaged after complete confluence was reached. Confluent plates were trypsinized and split, and the cells were cultured until the fourth passage was reached.

### Matrigel Tube Formation Assay

To initially determine if SsnB inhibited pro-angiogenic cell functions, a tube formation assay with Matrigel was performed. Growth factor reduced Matrigel (BD Biosciences, Bedford, MA) was added to the wells of a 96 well polystyrene culture plate and incubated at 37°C for 30 minutes. Cells (HUVECs, HCAECs, or HMVECs at passage 2 to 4) were added to each well to reach a final number of 20,000 cells per well. SsnB was added to the wells at a concentration of 1, 10, or 100 µM. Endothelial cell medium with DMSO (0.1%) was used as a vehicle control. Each group contained 4 replicates. The plates were placed in an incubator for 4 h. During the incubation, the endothelial cells formed elongated structures called cords, also known as tubes. After 4 h, neutral buffered formalin was added to fix the cells. Pictures of three non-overlapping fields were taken from each well. The lengths of single cell endothelial cords were measured with Image-Pro Plus (Media Cybernetics, Silver Spring, MD), and the sum of tube lengths for each well was determined. The average total length and standard deviation for each group were determined, and the appropriate statistical tests (ANOVA and Newman-Keuls) were completed. The tube formation assay was replicated three times for both HUVECs and HCAECs. The assay was also repeated with cardiac HMVECs.

### Cell Viability

A Live/Dead assay (Invitrogen, Eugene, OR) was utilized to investigate the effect of SsnB on cell viability. The Matrigel tube formation experiment was repeated with HUVECs in chamber slides at a concentration of 20,000 cells per well. The cells were treated with SsnB (1, 10, or 100 µM) or Vehicle Control (0.1% DMSO) as described above. After four hours of incubation, the slides were removed. A chamber slide containing HUVECs treated with 70% methanol for 30 minutes was used as a control for dead cell staining. The slides were aspirated and washed with PBS, and EthD-1 and calcein AM were added to each well. The plates were incubated in the dark, and images were taken with a light microscope at 10X magnification.

### Transwell Insert Cell Migration Assay

The cell invasion assay was performed with a Transwell insert system (6.5 mm diameter inserts with 8.0 µm pores in a polycarbonate membrane situated in wells of 24 well polystrene, tissue culture-treated plates, Corning Incorporated, Corning, NY). The Transwell inserts were coated with 0.1% gelatin for 30 min and incubated in low serum medium for 1 h. Cultured HUVECs were trypsinized and then resuspended in low serum medium (0.5% fetal bovine serum without endothelial cell mitogen), and added at 50,000 cells per insert. The cells were allowed to adhere to the inserts for 30 min. Next, various concentrations of SsnB (0.0001, 0.001, 0.01, 0.1, 1, 10, and 100 µM) or vehicle control (0.1% DMSO) were added to the Transwell inserts. After 30 min, the medium in the lower chamber for the experimental groups was replaced with low serum medium containing 10 ng/ml VEGF, establishing a chemoattractant gradient between the top insert and lower chamber. For the negative control group, the medium was replaced with low serum medium (0.5% fetal bovine serum). The plates were incubated for 8 h at 37°C. During the incubation, the cells migrated through the pores of the Transwell insert towards the lower chamber. At the end of this period, cells on the upper surface of the insert were removed, and migrated cells on the bottom side were fixed in formalin and stained with Hoechst dye (a fluorescent nuclear stain). The filter inserts were removed from the wells and mounted on glass slides. Cells were counted from four random fields observed with a 10X objective lens. The cell migration experiments were repeated three times at high SsnB concentrations (0.1, 1, 10, 100 µM) and one time at low SsnB concentrations (0.0001, 0.001, 0.01, 0.1 µM) for HUVECs.

### Cell Cycle Analysis

Cell cycle analysis was performed using propidium iodide staining and flow cytometry. HUVECs (approximately 75% confluent) cultured in 6 well polystyrene culture plates coated with 0.1% gelatin were serum starved for 24 hours in low serum medium containing 0.5% fetal bovine serum and no endothelial cell mitogen to synchronize the cells in the G0/G1 phase. After 24 hours, the low serum medium was replaced with treatments of either 100 µM SsnB or vehicle control (0.1% DMSO) diluted in complete growth medium (10% fetal bovine serum with endothelial cell mitogen) in triplicate. The treated cells were incubated for 24, 30, and 36 hours. After incubation, the cells were trypsinized, transferred to 5 ml polysytrene round bottom tubes (12×75 mm), and centrifuged. The medium was aspirated, and the cells were washed with PBS. After fixing the cells with ice-cold 70% ethanol for 15 min, the cells were centrifuged and stained with propidium iodide for 30 min. The samples were then analyzed with a flow cytometer (Beckman Coulter FC500). The unstained and stained cell groups were used to calibrate the settings on the flow cytometer. The data were collected and analyzed with ModFit software.

### Microarray Analysis

Confluent plates (100×20 mm polystyrene, tissue culture-treated petri plates coated with 0.1% gelatin, 75% confluent) of HUVECs and HCAECs (four plates for HUVECs, n = 2, and six plates for HCAECs, n = 3) were chosen for the microarray experiments. Half of the plates received complete growth medium (10% fetal bovine serum with endothelial mitogen) containing vehicle control (0.1% DMSO), and the remaining plates received complete growth medium containing 100 µM SsnB. The plates were incubated for 24 h to allow SsnB to have an effect on cellular gene expression. Following incubation, RNA isolation was completed with the RNeasy Mini kit from Qiagen. The cells were lysed, and RNA was isolated by using the RNeasy spin columns and following the protocol provided by Qiagen. Purified RNA was sent to the Medical University of South Carolina Proteogenomics Facility for microarray analysis. The GeneChip Human Genome U133 Plus 2.0 Array was utilized to track changes in gene expression due to SsnB treatment. Complete data was uploaded to the NCBI Gene Expression Omnibus database (accession number GSE44598).

### Real Time RT-PCR

After a careful analysis of the microarray data, key genes (CDC6, CCNE2, KITLG, ALDH3A1, CCNB1, CDC2, HMMR, DIAPH3, ANLN, and CDKN3) were chosen for quantitative real-time PCR (qRT-PCR) to verify the gene expression results. HCAECs and HUVECs were exposed to SsnB or vehicle control for 24 h (as previously described in the microarray section). For RT-PCR, RNA was isolated from the cells with the RNeasy Mini kit as described previously. After forward and reverse primer kits (Qiagen, Valencia, CA) were selected, the RNA was amplified. The one-step RT-PCR reactions were completed on the BioRad iCycler thermal cycler system in the Instrumentation Resource Facility at the USC School of Medicine. The expression levels were normalized, and RNA levels were quantified.

### Data Analysis

Microarray data analysis, including data normalization (robust multi-array average), identification of differentially expressed genes (comparative analyses with dChip software), and heat map construction, was carried out to determine how SsnB affects gene expression and affected pathways (n = 2 for HUVECs and n = 3 for HCAECs). After a careful analysis of the microarray data, several key genes were chosen for qRT-PCR to verify the gene expression results. The genes were chosen based on the following criteria: fold change ≥2, p<0.05, unpaired t-test with a false discovery rate approximating 0%, appearing in both data sets (HUVECs and HCAECs), and gene function relating to cell proliferation and/or angiogenesis. For qRT-PCR, the expression levels were normalized to the housekeeping gene GAPDH, and RNA levels were compared between groups with the ΔΔC_t_ method.

### Chick Chorioallantoic Membrane (CAM) Assay

Fresh fertilized Bovans eggs were obtained from Clemson University and stored at 4°C for no longer than one week. Methylcellulose discs were created with a solution of 0.5% methylcellulose (diluted in sterile deionized water). A methylcellulose solution of 100 micromolar SsnB was made by adding 1.1 microliters of stock SsnB (93 mM) to 1 ml 0.5% methylcellulose. A vehicle control solution was created by adding 1 microliter of dimethyl sulfoxide (DMSO) to 1 ml 0.5% methylcellulose. Discs were created by placing 20 microliter droplets of SsnB or vehicle control solution onto polytetrafluoroethylene tape (to minimize adherence during drying) and allowed to dry under a laminar flow hood. The fertilized eggs were placed in a humidified egg incubator with forced air circulation at 37°C. The eggs were automatically rotated every three hours. After 4 days of incubation, the eggs were removed and cracked. Chick embryos with intact yolks were placed in 100 mm petri plates. Each petri plate was placed in a weigh boat containing 13 ml Moscona's buffer and covered with cellophane wrap with two holes for air circulation. The plates were placed in a water-jacketed incubator (37C, 5% CO_2_) for 2 days. After 2 days of incubation, the methylcellulose discs containing either SsnB or vehicle control were placed on top of the CAM of each embryo in the right upper quadrant in an area near the allantoic vessels. After 2 additional days of incubation, the CAMs were examined and photographed with a dissecting stereomicroscope. Images of areas containing the discs and non-treated regions were analyzed with Image-Pro Plus software. Blood vessel length and branching number were quantified to measure the effect of SsnB on angiogenesis. The total vessel length was measured for areas containing the discs and for non-treated areas of the same size. Normalized vessel length was calculated by dividing the total length values of the treated regions by those of the non-treated regions. The same process was repeated for measuring the branching number. Each group contained seven embryos (calculated with PS 3.0.5 software, effect size of 50%, standard deviation of 30%, statistical power of 0.8, and α = 0.05). The CAM assay was completed two times.

## Results

### SsnB inhibits endothelial cell tube formation and cell migration

**Figure 1:**
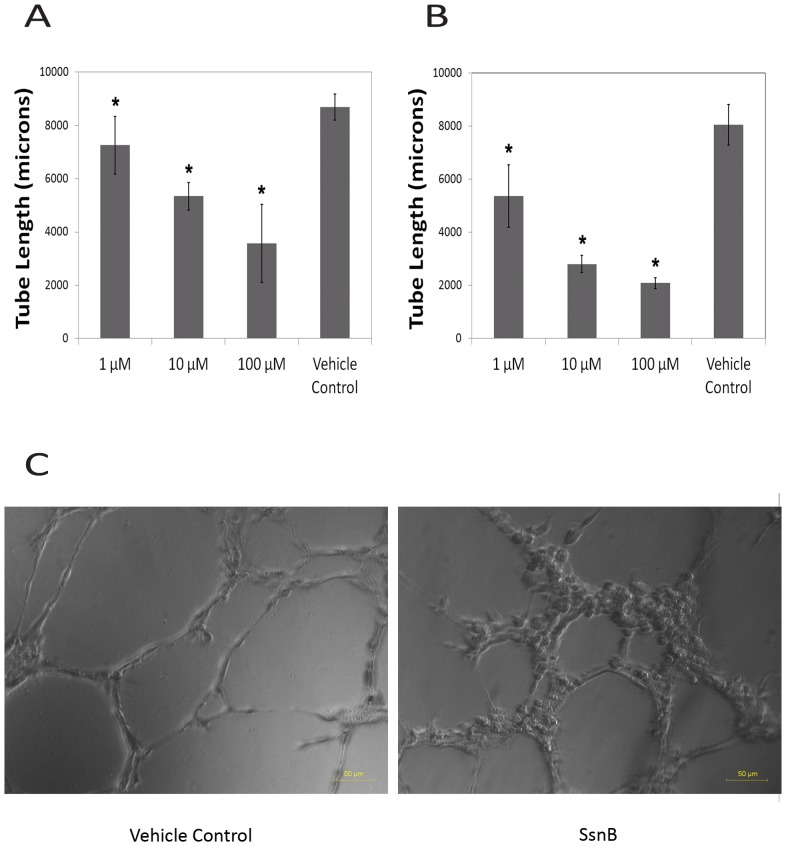
SsnB inhibits endothelial cell tube formation on Matrigel. A. Total tube length as a function of SsnB concentration in HUVECs. B. Total tube length as a function of SsnB concentration in HCAECs. *p<0.05 vs. vehicle control, Newman-Keuls test. C. Representative micrographs demonstrating tube formation in HUVECs (left – vehicle control, right – 100 µM SsnB).

**Figure 2:**
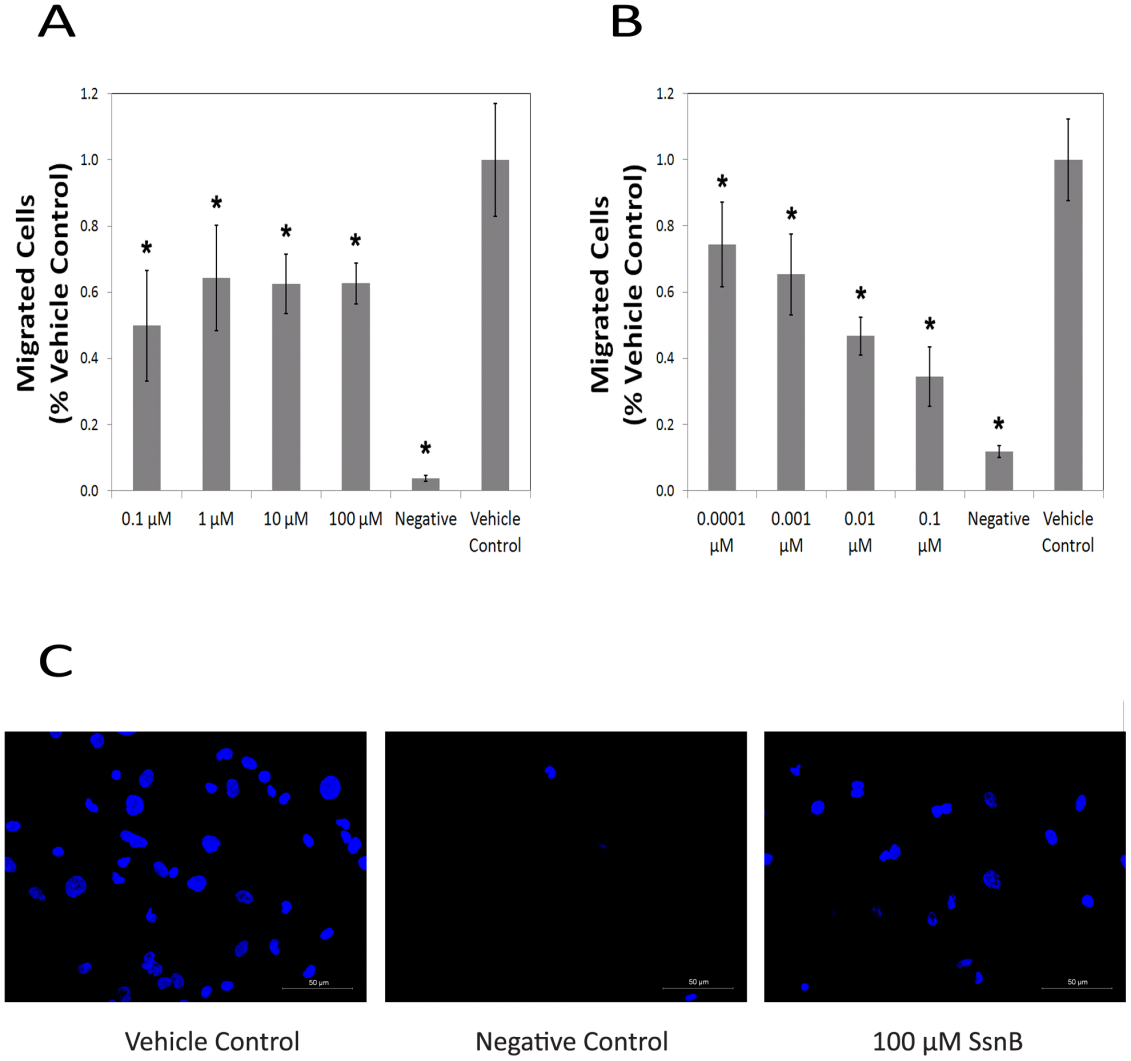
SsnB inhibits endothelial cell migration. A. Migrated cells as a function of SsnB concentration (0.1 to 100 µM). B. Migrated cells as a function of SsnB concentration (0.0001 to 0.1 µM) shows a dose-dependent response. *p<0.05 vs. vehicle control, Newman-Keuls test. C. Representative micrographs demonstrating cell migration (left – vehicle control, center – negative control, right – 100 µM SsnB).

Figures 1 and 2 show representative results from the tube formation and cell migration assays with HUVECs. SsnB treatment resulted in a dose-dependent inhibition of HUVEC tube formation at concentrations between 1 and 100 μM (p<0.05, Newman-Keuls test). SsnB also demonstrated a dose-dependent inhibition of VEGF-induced cell migration at concentrations between 0.0001 and 0.1 μM (p<0.05), which leveled off between 0.1 and 100 μM (p<0.05). Experiments with HCAECs also demonstrated a dose-dependent inhibition (between 1 and 100 µM) of endothelial cell tube formation on the substrate Matrigel (Figure 1B). A dose-dependent inhibition of tube formation was also seen with cardiac HMVECs (see Figure S1). Results from the live/dead assay with HUVECs showed that SsnB had no effect on cell viability at the concentrations used. Cells treated with SsnB or vehicle control showed positive staining for live cells, but no staining for dead cells (red fluorescence). These data suggest that SsnB is able to inhibit endothelial cell morphogenesis and that cell migration, a crucial process in angiogenesis, may play a role.

### SsnB arrests endothelial cells in the G1 phase of the cell cycle

**Figure 3:**
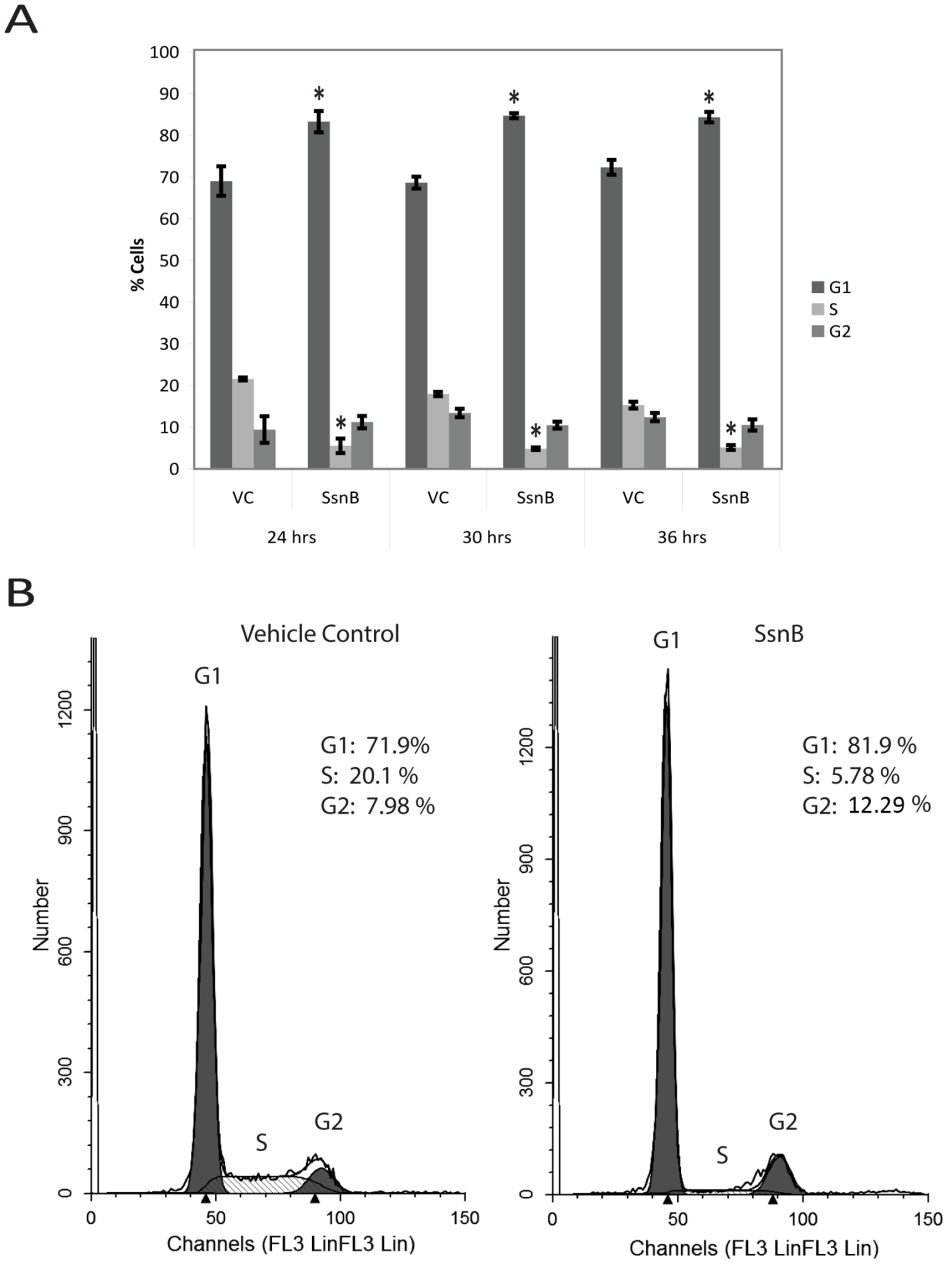
SsnB arrests endothelial cells in the G1 phase of the cell cycle. A. After 24, 30, and 36 hours of treatment, 100 µM SsnB decreased the percentage of cells in the S phase and increased the percentage of cells in the G1 phase. *p<0.005 vs. corresponding vehicle control, Newman-Keuls test. B. Representative cell cycle data demonstrating the increase in G1 cell percentage and decrease in S phase cell percentage after 24 hours of SsnB treatment (left – vehicle control, right – SsnB).

Figure 3 depicts representative results from the cell cycle experiments. In comparison to vehicle controls, flow cytometric cell cycle analysis of HUVECs treated with SsnB showed an increase of cells in the G1 phase and a decrease of cells in the S phase after 24, 30, and 36 hours of treatment. In untreated cells, the percentage of cells in S phase decreased and the percentage in G2/M increased from 24 to 36 hours after addition of growth medium, as expected once cells begin to re-enter and move through the cell cycle. Overall, these results imply that endothelial cells are being arrested in the G0/G1 phase, suggesting an inhibition of cell proliferation, an important step in angiogenesis.

### SsnB changes the expression of genes associated with cell cycle and cell proliferation

Microarray experiments demonstrated differential expression of several hundred genes in response to SsnB exposure (916 genes for HUVECs and 356 genes for HCAECs, fold change ≥2, p<0.05, unpaired t-test with a false discovery rate approximating 0%). Table S1 and Table S2 present the results of the gene function enrichment analysis for HUVECs and HCAECs, respectively. Overall, microarray data from both cell types showed significant overlap, including genes in pathways associated with cell proliferation, cytoskeleton, chemotaxis, and cell cycle, all areas implicated in angiogenesis. These results are consistent with the data obtained from the cell migration and cell cycle functional studies. From this microarray study, it is clear that SsnB regulates genes involved in angiogenic processes in HUVECs and HCAECs.

### Real Time RT-PCR

Following microarray data analysis, key genes (listed in Table 1) were chosen for verification with real time RT-PCR. Cyclin E2 (CCNE2) and Cell division cycle 6 (CDC6) are regulatory proteins that control cell cycle progression through the G1/S checkpoint. Both CCNE2 and CDC6 were downregulated in the microarray data. qRT- PCR confirmed that gene expression of CCNE2 and CDC6 was downregulated after SsnB exposure to 64% and 35% of controls, respectively, for HUVECs and to 57% and 14% of controls, respectively, for HCAECs. Kit-Ligand (KITLG), also known as stem cell factor, is a protein involved in the differentiation and growth of stem cells. Aldehyde dehydrogenase 3 family, member A1 (ALDH3A1) is a protein involved in the aryl hydrocarbon receptor pathway. These genes were chosen for further study because they were highly upregulated by SsnB treatment in the microarray data set. In HUVECs, qRT-PCR analysis demonstrated that KITLG and ALDH3A1 expression was upregulated to 400% and 1280% of controls, respectively. In HCAECs, KITLG and ALDH3A1 expression was upregulated to 260% and 4620% of controls, respectively. The microarray data was supported in both data sets.

**Table 1:** Comparison of RT-PCR and Microarray Results.

	HUVECs			HCAECs		
Gene	2^ΔΔCt^ Value	Microarray Value	Microarray p-value	2^ΔΔCt^ Value	Microarray Value	Microarray p-value
KITLG	4.0	6.2	0.02795	2.6	2.1	0.00010
ALDH3A1	12.8	7.7	0.00609	46.2	8.8	0.00001
HMMR	0.24	0.11	0.03073	0.062	0.20	0.00006
DIAPH3	0.19	0.34	0.03248	0.26	0.38	0.00019
ANLN	0.20	0.09	0.00900	0.13	0.11	0.000001
CDKN3	0.18	0.19	0.01471	0.22	0.18	0.00018
CCNB1	0.40	0.19	0.02304	0.13	0.16	0.00003
CDC2	0.14	0.08	0.00440	0.17	0.20	0.00004
CDC6	0.35	0.23	0.01527	0.14	0.18	0.00185
CCNE2	0.64	0.24	0.01814	0.57	0.38	0.00180

Fold expression change vs. vehicle control.

### SsnB inhibits ex vivo angiogenesis in the CAM assay

**Figure 4:**
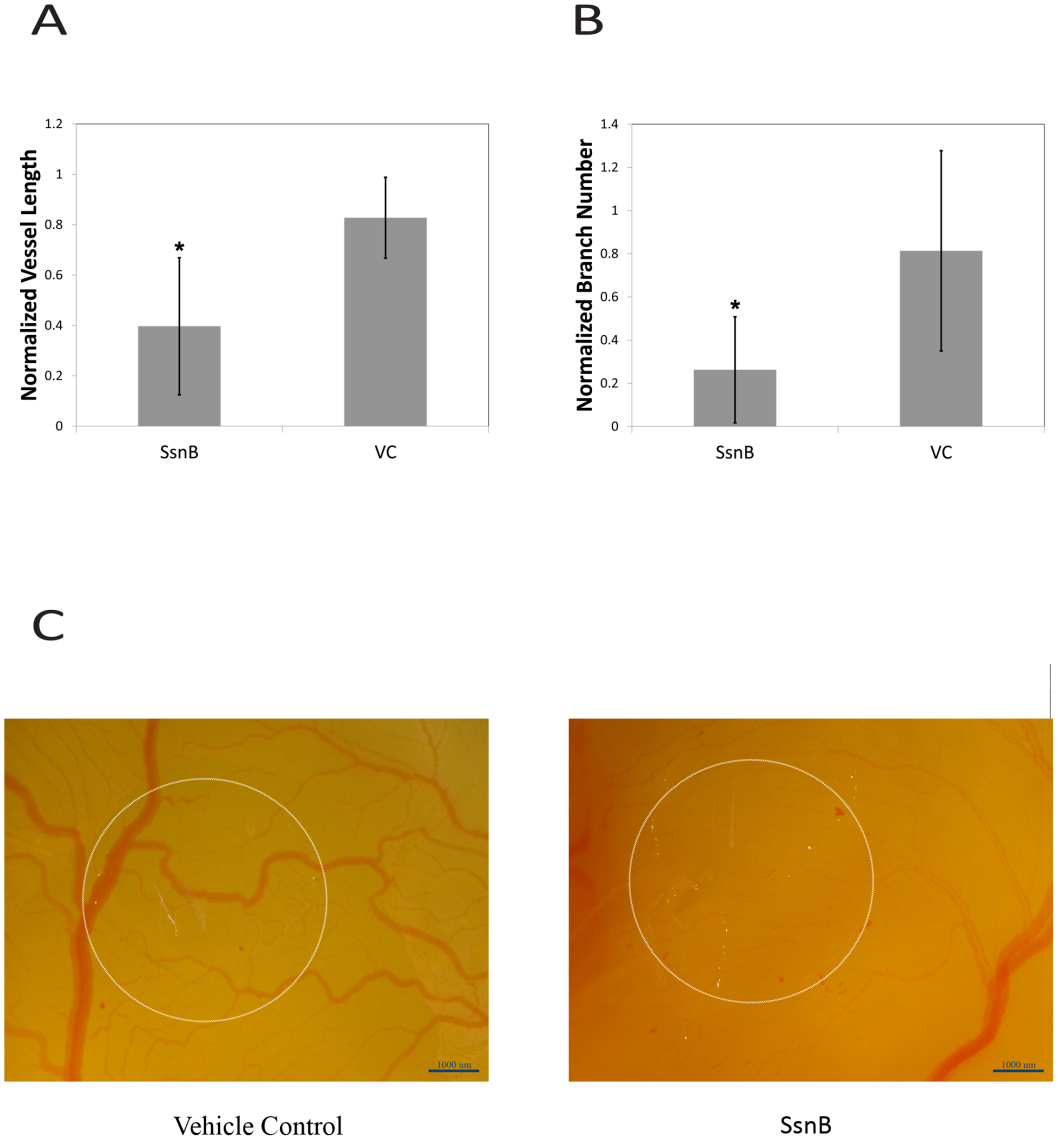
SsnB inhibits ex vivo angiogenesis in the CAM assay. A. 100 µM SsnB decreased normalized total blood vessel length. B. 100 µM SsnB decreased normalized branching number. *p<0.05 vs. vehicle control, Newman-Keuls test. C. Representative micrographs demonstrating blood vessels in the CAM assay (left – vehicle control, right – 100 µM SsnB, circles show approximate position of methylcellulose discs).

The results from the CAM assay are depicted in Figure 4. Overall, SsnB treatment resulted in a significant decrease in normalized blood vessel length compared to the vehicle control group (ANOVA, p<0.05). In addition to reducing total blood vessel length, SsnB treatment also resulted in a significant decrease in the normalized branch number. Compared to the vehicle control, SsnB decreased the number of points where blood vessels branched into two or more separate vessels. Representative images of the CAM from the SsnB and vehicle control groups after two days of treatment are shown in figure 4. The methylcellulose discs released SsnB or vehicle control and were still present at the end of the experiment, as complete biodegradation did not occur. The transparency of the methylcellulose discs allowed easy imaging of the vascular network underneath the discs. Images were taken of regions of the CAM that received methylcellulose discs as well as regions that did not receive discs. For each embryo, the blood vessel length and branch number were measured for both treated and non-treated areas. A normalized value was calculated by dividing the treated areas by the non-treated areas. This approach allowed us to compensate for natural variations in vascular density among embryos that normally occur even in the absence of angiogenic inhibitors or stimulators. Overall, the SsnB-treated group showed fewer blood vessels, reduced vessel lengths, and fewer branches. The methylcellulose discs with SsnB not only inhibited angiogenesis in the area underneath the disc, but also demonstrated inhibition in the immediate area surrounding the discs. Compared to the SsnB group, the vehicle control group exhibited greater vessel lengths, more numerous vessels, and more vascular branching. In addition, SsnB had no effect on the viability or morphology of the embryos relative to the vehicle control.

## Discussion

Pathological angiogenesis is associated with many disorders, including rheumatoid arthritis, diabetic retinopathy, and psoriasis. Recent research has shown that inhibiting angiogenesis may prove to be an effective therapeutic option in treating these disorders. In the present study, potential anti-angiogenic effects of SsnB were demonstrated in the *ex vivo* CAM assay and in several *in vitro* functional assays with endothelial cells, including the Matrigel tube formation assay and the Transwell insert cell migration assay. SsnB was also shown to arrest endothelial cells in the G1 phase of the cell cycle. The cellular functions tested in these assays represent key steps in angiogenesis that are inhibited by SsnB, including cell proliferation, cell migration, and chemotaxis. To further investigate the mechanism of action for SsnB, we utilized microarray analysis to examine how SsnB affected gene expression in endothelial cells. After SsnB exposure, genes associated with cell proliferation, cell cycle, chemotaxis, and the cytoskeleton were differentially regulated in both HUVECs and HCAECs. Our data suggest that SsnB alters gene expression in these pro-angiogenic pathways.

Targeting endothelial cell proliferation has shown potential in the area of angiogenesis inhibition. Endothelial cell proliferation may be inhibited in numerous ways, including the downregulation of cell cycle regulatory proteins [10]. Cyclins and cyclin dependent kinases are regulatory proteins that control progression through the cell cycle by regulating specific cell cycle checkpoints. Cyclins help activate cyclin dependent kinases, which phosphorylate downstream proteins that allow cells to progress through these checkpoints. Cyclin E2 (CCNE2) and Cell division cycle 6 (CDC6) are regulatory proteins that control progression through the G1/S checkpoint. CDC6 regulates DNA replication, and cyclin E2 activates cyclin-dependent kinase 2 [11], [12]. Downregulation of CCNE2 and CDC6 will trap cells at the G1/S checkpoint and prevent endothelial cells from initiating DNA replication. Genes encoding the cell cycle regulatory proteins, Cyclin E2 (CCNE2) and Cell division cycle 6 (CDC6), were both downregulated by SsnB treatment. Overall, our data suggest that SsnB downregulates the cell cycle regulatory proteins CCNE2 and CDC6, potentially trapping cells in the G1 phase and hindering cell proliferation, an important step in angiogenesis [13]–[16].

Our data show that SsnB affects the gene expression of additional cell cycle regulatory proteins, including cyclin B1 (CCNB1) and cyclin dependent kinase 1 (CDC2). CCNB1 and CDC2 are regulatory proteins that control progression through the G2/M checkpoint. Downregulation of CCNB1 and CDC2 will trap cells at the G2/M checkpoint and prevent endothelial cells from entering the final stages of cell division. Table 1 demonstrates that CCNB1 and CDC2 were both downregulated by SsnB. In addition to preventing cell progression through the G1 checkpoint, SsnB may also prevent progression through the G2/M checkpoint by downregulating these additional cell cycle proteins [13]–[16]. This effect is less readily seen due to blockage at the G1 checkpoint in synchronized cells.

*Sparganium stoloniferum* has long been used in Traditional Chinese Medicine for the treatment of cancer and seizures. Major chemical components from the stem and rhizome, including flavonoids, saponins, and phenylpropanoids, are responsible for these therapeutic effects [17]. Anti-tumor agents often exhibit anti-angiogenic effects, among other cancer suppressing properties. Inhibiting angiogenesis cuts tumors off from the vasculature, limiting growth and metastasis [18]. Previously published research has shown that extracts derived from *Sparganium stoloniferum* have demonstrated potent anti-cancer effects. Isolated chemical compounds from this herb, including a sucrose ester, a phenylpropanoid glycerol, carboxylic acid esters, and a phenylpropanoid glycoside, demonstrated anti-tumor activities [2], [19]. In addition, the polyphenolic structure of SsnB also reveals much about its potential therapeutic effects. Many plant-derived polyphenols have demonstrated anti-inflammatory and anti-angiogenic properties. Quercetin and resveratrol, both polyphenols isolated from red wine, have demonstrated anti-inflammatory and anti-angiogenic properties. In addition, oleuropein and hydroxytyrosol, both derived from virgin olive oil, reduce angiogenesis by inhibiting matrix metalloproteinase-9 (MMP-9) and cyclooxygenase-2 (COX-2) [20]. Emodin, another plant-derived polyphenol similar to SsnB, has also been shown to inhibit angiogenesis by targeting endothelial cell proliferation [21]. Emodin causes a downregulation of CCNB1 and CDC2 and a cell cycle arrest at the G2/M phase [22].

In addition to its effects on the cell cycle and cell proliferation, SsnB also affects other key steps of angiogenesis, including cell migration and chemotaxis. The microarray data for both HUVECs and HCAECs demonstrates an enrichment of genes associated with both pathways, including diaphanous homolog 3 (DIAPH3), hyaluronan-mediated motility receptor (HMMR), and anillin (ANLN), an actin binding protein. Furthermore, it is also important to investigate potential upstream pathways that lead to the alterations in gene expression seen in the microarray data. Previously published data have shown that SsnB can suppress the NF-κB pathway, and prior studies have demonstrated a link between NF-κB signaling and expression of cell cycle proteins. Genes for these regulatory proteins, such as CCNE2, CDC6, CCNB1, and CDC2, contain binding sites for NF-κB. SsnB may suppress the NF-κB pathway and cause a subsequent downregulation of the cell cycle regulatory proteins CCNE2 and CDC6. SsnB has also been shown to inhibit the MAPK pathways, including JNK, ERK, and p38 signaling, which all affect gene expression and cell proliferation [23]–[27]. We need to explore these relationships as potential mechanisms of angiogenesis inhibition.

Furthermore, the CAM assay allowed us to examine the effects of SsnB in an *ex vivo* system involving additional cell types beyond endothelial cells. Multiple cell types are involved in angiogenesis, including endothelial cells and supporting cells, such as pericytes, smooth muscle cells, macrophages, and myofibroblasts. Since our initial *in vitro* experiments only involved endothelial cells, we utilized the CAM assay to address the multicellular nature of angiogenesis. We are particularly interested in how SsnB affects macrophages. In the CAM assay, SsnB inhibited blood vessel branching. It has been demonstrated that macrophage production of matrix metalloproteinase-9 (MMP-9) plays an important role in vascular branching during neovascularization [28]. Prior research has shown that SsnB blocks TLR-2 and TLR-4 mediated inflammation in human and murine macrophages[3]. Furthermore, TLR signaling activates MMP production in macrophages [29]. We hypothesize that SsnB blocks TLR signaling and subsequently inhibits macrophage MMP-9 production required for blood vessel branching. Additional experiments could examine this relationship between SsnB, TLR signaling, macrophage production of MMP-9, and vascular branching.

Our current study with HUVECs and HCAECs demonstrates that SsnB inhibits angiogenic processes in functional assays, including the Matrigel tube assay and the Transwell insert cell migration assay, and that it downregulates multiple genes involved in pro-angiogenic pathways as shown by microarray analysis. We have also shown that SsnB inhibits *ex vivo* angiogenesis in the CAM assay. Future work with SsnB will reveal more about its mechanism of action, including cytoskeletal changes and possible binding sites. Overall, SsnB has proven to be a powerful bioactive compound with a multitude of useful therapeutic effects, including anti-angiogenesis and anti-inflammation.

## Supporting Information

Figure S1 **SsnB inhibits endothelial cell tube formation on Matrigel.** Total tube length as a function of SsnB concentration in HMVECs, *p<0.05 vs. vehicle control, Newman-Keuls test.
(TIFF)

Table S1 **Gene function enrichment analysis for HUVECs in response to SsnB treatment.**
(DOCX)

Table S2 **Gene function enrichment analysis for HCAECs in response to SsnB treatment.**
(DOC)
