# Supplementary figures and images for: Sparstolonin B Inhibits Pro-Angiogenic Functions and Blocks Cell Cycle Progression in Endothelial Cells
Source: PLoS One. 2013 Aug 5;8(8):e70500. doi: 10.1371/journal.pone.0070500 (PMC3734268; doi:10.1371/journal.pone.0070500)

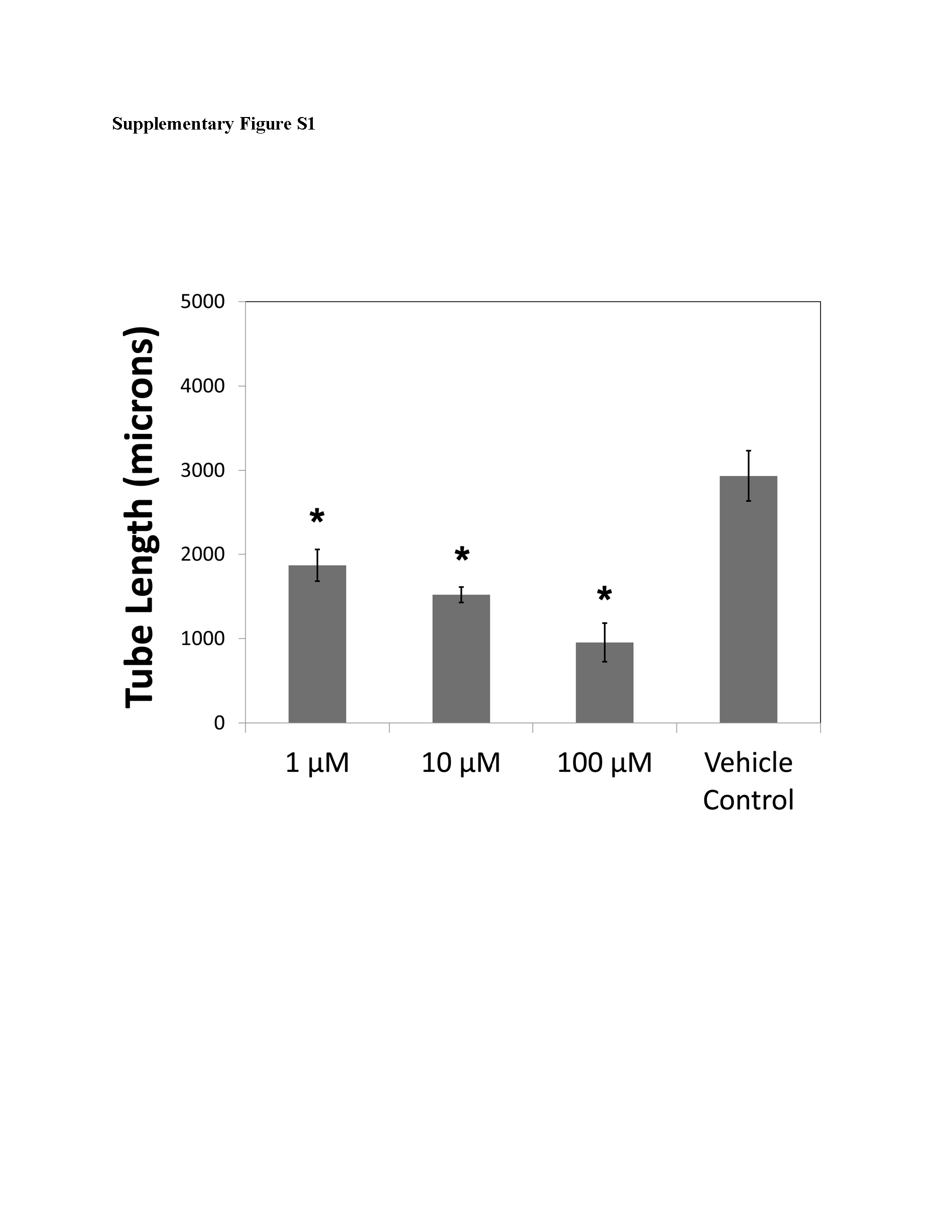

Supplement: Figure S1 — SsnB inhibits endothelial cell tube formation on Matrigel. Total tube length as a function of SsnB concentration in HMVECs, *p<0.05 vs. vehicle control, Newman-Keuls test. (TIFF) [file pone.0070500.s001.tiff]
