# Supplementary material for: Sparstolonin B Inhibits Pro-Angiogenic Functions and Blocks Cell Cycle Progression in Endothelial Cells
Source: PLoS One. 2013 Aug 5;8(8):e70500. doi: 10.1371/journal.pone.0070500 (PMC3734268; doi:10.1371/journal.pone.0070500)
Supplement: Table S1 — Gene function enrichment analysis for HUVECs in response to SsnB treatment. (DOCX) [file pone.0070500.s002.docx]

**Supplementary Table S1**

| ***Gene Ontology*** | | | | | |
| --- | --- | --- | --- | --- | --- |
| C1 | C2 | C3 | C4 | P-value | Term Name |
| 118 | 759 | 1470 | 15432 | 0 | adenyl nucleotide binding |
| 114 | 759 | 1386 | 15432 | 0 | ATP binding |
| 203 | 759 | 3326 | 15432 | 0.000397 | cell communication |
| 123 | 759 | 772 | 15432 | 0 | cell cycle |
| 15 | 759 | 75 | 15432 | 0.000007 | cell cycle arrest |
| 13 | 759 | 48 | 15432 | 0.000001 | cell cycle checkpoint |
| 64 | 759 | 258 | 15432 | 0 | cell cycle phase |
| 107 | 759 | 653 | 15432 | 0 | cell cycle process |
| 49 | 759 | 199 | 15432 | 0 | cell division |
| 54 | 759 | 588 | 15432 | 0.000013 | cell proliferation |
| 576 | 759 | 10922 | 15432 | 0.000936 | cellular process |
| 16 | 759 | 130 | 15432 | 0.000926 | chemotaxis |
| 38 | 759 | 286 | 15432 | 0 | chromosomal part |
| 44 | 759 | 337 | 15432 | 0 | chromosome |
| 13 | 759 | 43 | 15432 | 0 | chromosome segregation |
| 16 | 759 | 48 | 15432 | 0 | chromosome, pericentric region |
| 46 | 759 | 543 | 15432 | 0.000335 | cytoskeletal part |
| 66 | 759 | 897 | 15432 | 0.000881 | cytoskeleton |
| 47 | 759 | 439 | 15432 | 0.000001 | cytoskeleton organization and biogenesis |
| 17 | 759 | 119 | 15432 | 0.000121 | cytoskeleton-dependent intracellular transport |
| 57 | 759 | 711 | 15432 | 0.000271 | DNA metabolic process |
| 25 | 759 | 244 | 15432 | 0.000614 | DNA repair |
| 29 | 759 | 189 | 15432 | 0 | DNA replication |
| 16 | 759 | 96 | 15432 | 0.000033 | DNA-dependent DNA replication |
| 15 | 759 | 67 | 15432 | 0.000002 | interphase |
| 14 | 759 | 63 | 15432 | 0.000005 | interphase of mitotic cell cycle |
| 107 | 759 | 1541 | 15432 | 0.000199 | intracellular non-membrane-bound organelle |
| 66 | 759 | 897 | 15432 | 0.000881 | kinase activity |
| 57 | 759 | 214 | 15432 | 0 | M phase |
| 50 | 759 | 168 | 15432 | 0 | M phase of mitotic cell cycle |
| 25 | 759 | 202 | 15432 | 0.000037 | microtubule |
| 16 | 759 | 113 | 15432 | 0.000209 | microtubule associated complex |
| 37 | 759 | 290 | 15432 | 0 | microtubule cytoskeleton |
| 15 | 759 | 63 | 15432 | 0.000001 | microtubule cytoskeleton organization and biogenesis |
| 14 | 759 | 83 | 15432 | 0.000089 | microtubule motor activity |
| 17 | 759 | 105 | 15432 | 0.000027 | microtubule-based movement |
| 31 | 759 | 186 | 15432 | 0 | microtubule-based process |
| 48 | 759 | 166 | 15432 | 0 | mitosis |
| 58 | 759 | 226 | 15432 | 0 | mitotic cell cycle |
| 19 | 759 | 156 | 15432 | 0.000367 | motor activity |
| 75 | 759 | 873 | 15432 | 0.000003 | negative regulation of biological process |
| 71 | 759 | 806 | 15432 | 0.000002 | negative regulation of cellular process |
| 24 | 759 | 185 | 15432 | 0.000025 | negative regulation of progression through cell cycle |
| 107 | 759 | 1541 | 15432 | 0.000199 | non-membrane-bound organelle |
| 140 | 759 | 2090 | 15432 | 0.000093 | nucleotide binding |
| 14 | 759 | 91 | 15432 | 0.000225 | phosphoinositide-mediated signaling |
| 319 | 759 | 5058 | 15432 | 0 | protein binding |
| 134 | 759 | 1797 | 15432 | 0.000001 | purine nucleotide binding |
| 8 | 759 | 37 | 15432 | 0.000589 | Ras GTPase activator activity |
| 74 | 759 | 493 | 15432 | 0 | regulation of cell cycle |
| 217 | 759 | 3609 | 15432 | 0.000529 | regulation of cellular process |
| 9 | 759 | 41 | 15432 | 0.000245 | regulation of cyclin-dependent protein kinase activity |
| 14 | 759 | 46 | 15432 | 0 | regulation of mitosis |
| 74 | 759 | 490 | 15432 | 0 | regulation of progression through cell cycle |
| 31 | 759 | 282 | 15432 | 0.000042 | response to DNA damage stimulus |
| 31 | 759 | 304 | 15432 | 0.000157 | response to endogenous stimulus |
| 47 | 759 | 492 | 15432 | 0.000018 | response to external stimulus |
| 130 | 759 | 1996 | 15432 | 0.000545 | response to stimulus |
| 80 | 759 | 842 | 15432 | 0 | response to stress |
| 14 | 759 | 43 | 15432 | 0 | spindle |
| 16 | 759 | 130 | 15432 | 0.000926 | taxis |
| 76 | 759 | 1037 | 15432 | 0.000404 | transferase activity, transferring phosphorus-containing groups |
| ***Protein Domain*** | | | | | |
| C1 | C2 | C3 | C4 | P-value | Term Name |
| 4 | 542 | 9 | 12147 | 0.000781 | AIG1 family // 9.1E-85 |
| 6 | 542 | 16 | 12147 | 0.000098 | Cyclin, C-terminal // 4.0E-41 |
| 12 | 542 | 41 | 12147 | 0.000001 | Kinesin, motor region // 4.1E-121 |
| ***Pathway*** | | | | | |
| C1 | C2 | C3 | C4 | P-value | Term Name |
| 31 | 180 | 112 | 2918 | 0 | Cell_cycle_KEGG // GenMAPP |
| 21 | 180 | 69 | 2918 | 0 | DNA_replication_Reactome // GenMAPP |
| 19 | 180 | 90 | 2918 | 0.000004 | G1_to_S_cell_cycle_Reactome // GenMAPP |
| 25 | 180 | 195 | 2918 | 0.000429 | Smooth_muscle_contraction // GenMAPP |

C1: number of genes in a cluster or list that have this annotation term

C2: number of annotated genes in this cluster or list

C3: number of all genes on array that have this annotation term

C4: number of all annotated genes on array

P-value: binomial approximated p-value for hypergeometric distribution
