# Supplementary material for: Sparstolonin B Inhibits Pro-Angiogenic Functions and Blocks Cell Cycle Progression in Endothelial Cells
Source: PLoS One. 2013 Aug 5;8(8):e70500. doi: 10.1371/journal.pone.0070500 (PMC3734268; doi:10.1371/journal.pone.0070500)
Supplement: Table S2 — Gene function enrichment analysis for HCAECs in response to SsnB treatment. (DOC) [file pone.0070500.s003.doc]

**Supplementary Table S2**

| ***Gene Ontology*** | | | | | |
| --- | --- | --- | --- | --- | --- |
| C1 | C2 | C3 | C4 | P-value | Term Name |
| 56 | 214 | 1470 | 15432 | 0 | adenyl nucleotide binding |
| 56 | 214 | 1386 | 15432 | 0 | ATP binding |
| 15 | 214 | 360 | 15432 | 0.000177 | ATPase activity |
| 13 | 214 | 311 | 15432 | 0.000463 | ATPase activity, coupled |
| 87 | 214 | 4602 | 15432 | 0.000477 | biopolymer metabolic process |
| 93 | 214 | 5029 | 15432 | 0.000574 | catalytic activity |
| 85 | 214 | 772 | 15432 | 0 | cell cycle |
| 13 | 214 | 48 | 15432 | 0 | cell cycle checkpoint |
| 51 | 214 | 258 | 15432 | 0 | cell cycle phase |
| 71 | 214 | 653 | 15432 | 0 | cell cycle process |
| 43 | 214 | 199 | 15432 | 0 | cell division |
| 27 | 214 | 588 | 15432 | 0 | cell proliferation |
| 51 | 214 | 2215 | 15432 | 0.000155 | cellular component organization and biogenesis |
| 179 | 214 | 10922 | 15432 | 0.000009 | cellular process |
| 32 | 214 | 286 | 15432 | 0 | chromosomal part |
| 35 | 214 | 337 | 15432 | 0 | chromosome |
| 19 | 214 | 377 | 15432 | 0.000002 | chromosome organization and biogenesis |
| 15 | 214 | 363 | 15432 | 0.000194 | chromosome organization and biogenesis (sensu Eukaryota) |
| 15 | 214 | 43 | 15432 | 0 | chromosome segregation |
| 18 | 214 | 48 | 15432 | 0 | chromosome, pericentric region |
| 6 | 214 | 34 | 15432 | 0.00001 | condensed chromosome |
| 30 | 214 | 543 | 15432 | 0 | cytoskeletal part |
| 31 | 214 | 897 | 15432 | 0.000003 | cytoskeleton |
| 28 | 214 | 439 | 15432 | 0 | cytoskeleton organization and biogenesis |
| 13 | 214 | 119 | 15432 | 0 | cytoskeleton-dependent intracellular transport |
| 4 | 214 | 30 | 15432 | 0.000877 | DNA damage response, signal transduction |
| 45 | 214 | 711 | 15432 | 0 | DNA metabolic process |
| 21 | 214 | 244 | 15432 | 0 | DNA repair |
| 30 | 214 | 189 | 15432 | 0 | DNA replication |
| 9 | 214 | 42 | 15432 | 0 | DNA-dependent ATPase activity |
| 18 | 214 | 96 | 15432 | 0 | DNA-dependent DNA replication |
| 4 | 214 | 26 | 15432 | 0.000517 | double-stranded DNA binding |
| 21 | 214 | 618 | 15432 | 0.000162 | hydrolase activity, acting on acid anhydrides |
| 21 | 214 | 615 | 15432 | 0.000152 | hydrolase activity, acting on acid anhydrides, in phosphorus-containing anhydrides |
| 12 | 214 | 67 | 15432 | 0 | interphase |
| 12 | 214 | 63 | 15432 | 0 | interphase of mitotic cell cycle |
| 140 | 214 | 7906 | 15432 | 0.000019 | intracellular |
| 108 | 214 | 5631 | 15432 | 0.000021 | intracellular membrane-bound organelle |
| 60 | 214 | 1541 | 15432 | 0 | intracellular non-membrane-bound organelle |
| 122 | 214 | 6494 | 15432 | 0.000008 | intracellular organelle |
| 62 | 214 | 2068 | 15432 | 0 | intracellular organelle part |
| 131 | 214 | 7364 | 15432 | 0.00005 | intracellular part |
| 47 | 214 | 214 | 15432 | 0 | M phase |
| 45 | 214 | 168 | 15432 | 0 | M phase of mitotic cell cycle |
| 108 | 214 | 5633 | 15432 | 0.000021 | membrane-bound organelle |
| 17 | 214 | 202 | 15432 | 0 | microtubule |
| 13 | 214 | 113 | 15432 | 0 | microtubule associated complex |
| 28 | 214 | 290 | 15432 | 0 | microtubule cytoskeleton |
| 12 | 214 | 63 | 15432 | 0 | microtubule cytoskeleton organization and biogenesis |
| 13 | 214 | 83 | 15432 | 0 | microtubule motor activity |
| 6 | 214 | 64 | 15432 | 0.000303 | microtubule organizing center |
| 13 | 214 | 105 | 15432 | 0 | microtubule-based movement |
| 25 | 214 | 186 | 15432 | 0 | microtubule-based process |
| 44 | 214 | 166 | 15432 | 0 | mitosis |
| 50 | 214 | 226 | 15432 | 0 | mitotic cell cycle |
| 13 | 214 | 156 | 15432 | 0 | motor activity |
| 60 | 214 | 1541 | 15432 | 0 | non-membrane-bound organelle |
| 21 | 214 | 583 | 15432 | 0.000073 | nucleoside-triphosphatase activity |
| 56 | 214 | 2090 | 15432 | 0.000001 | nucleotide binding |
| 101 | 214 | 3964 | 15432 | 0 | nucleus |
| 122 | 214 | 6495 | 15432 | 0.000008 | organelle |
| 44 | 214 | 1019 | 15432 | 0 | organelle organization and biogenesis |
| 62 | 214 | 2068 | 15432 | 0 | organelle part |
| 13 | 214 | 91 | 15432 | 0 | phosphoinositide-mediated signaling |
| 33 | 214 | 1302 | 15432 | 0.000564 | post-translational protein modification |
| 15 | 214 | 421 | 15432 | 0.000888 | protein serine/threonine kinase activity |
| 56 | 214 | 1797 | 15432 | 0 | purine nucleotide binding |
| 21 | 214 | 612 | 15432 | 0.000142 | pyrophosphatase activity |
| 43 | 214 | 493 | 15432 | 0 | regulation of cell cycle |
| 73 | 214 | 3609 | 15432 | 0.000246 | regulation of cellular process |
| 7 | 214 | 41 | 15432 | 0.000002 | regulation of cyclin-dependent protein kinase activity |
| 5 | 214 | 43 | 15432 | 0.00037 | regulation of DNA metabolic process |
| 13 | 214 | 46 | 15432 | 0 | regulation of mitosis |
| 43 | 214 | 490 | 15432 | 0 | regulation of progression through cell cycle |
| 24 | 214 | 282 | 15432 | 0 | response to DNA damage stimulus |
| 24 | 214 | 304 | 15432 | 0 | response to endogenous stimulus |
| 30 | 214 | 842 | 15432 | 0.000002 | response to stress |
| 13 | 214 | 219 | 15432 | 0.000015 | second-messenger-mediated signaling |
| 13 | 214 | 43 | 15432 | 0 | spindle |
| 28 | 214 | 1037 | 15432 | 0.00059 | transferase activity, transferring phosphorus-containing groups |
| 8 | 214 | 143 | 15432 | 0.000965 | ubiquitin-protein ligase activity |
| ***Protein Domain*** | | | | | |
| C1 | C2 | C3 | C4 | P-value | Term Name |
| 5 | 130 | 47 | 12147 | 0.000166 | AAA ATPase, central region // 2.2E-18 |
| 4 | 130 | 29 | 12147 | 0.00029 | BRCT // 9.8E-9 |
| 4 | 130 | 16 | 12147 | 0.00003 | Cyclin, C-terminal // 4.0E-41 |
| 4 | 130 | 31 | 12147 | 0.000373 | Cyclin, N-terminal domain // 3.6E-14 |
| 12 | 130 | 41 | 12147 | 0 | Kinesin, motor region // 4.1E-121 |
| 2 | 130 | 4 | 12147 | 0.000884 | Lysophospholipase, catalytic domain // 2.4E-63 |
| 2 | 130 | 3 | 12147 | 0.000501 | M-phase inducer phosphatase // 1.7E-30 |
| 6 | 130 | 10 | 12147 | 0 | MCM // 2.1E-66 |
| 2 | 130 | 4 | 12147 | 0.000884 | SMC protein, N-terminal // 3.7E-50 |
| ***Pathway*** | | | | | |
| C1 | C2 | C3 | C4 | P-value | Term Name |
| 27 | 60 | 112 | 2918 | 0 | Cell_cycle_KEGG // GenMAPP |
| 22 | 60 | 69 | 2918 | 0 | DNA_replication_Reactome // GenMAPP |
| 16 | 60 | 90 | 2918 | 0 | G1_to_S_cell_cycle_Reactome // GenMAPP |

C1: number of genes in a cluster or list that have this annotation term

C2: number of annotated genes in this cluster or list

C3: number of all genes on array that have this annotation term

C4: number of all annotated genes on array

P-value: binomial approximated p-value for hypergeometric distribution
